# Direct observation of an exposed blood vessel in a colonic diverticulum using ultrathin endoscopy

**DOI:** 10.1002/deo2.70032

**Published:** 2024-10-29

**Authors:** Kensuke Suzuki, Daisuke Kikuchi, Satoshi Yamashita

**Affiliations:** ^1^ Department of Gastroenterology Toranomon Hospital Branch Kanagawa Japan; ^2^ Department of Gastroenterology Toranomon Hospital Tokyo Japan

**Keywords:** bleeding diverticulum, endoscopic band ligation, observation of the diverticulum, sigmoid colon, ultrathin endoscopy

## Abstract

In recent years, cases of diverticular bleeding have become more common. Although identifying the bleeding diverticulum is difficult, it is even more difficult to identify the exposed blood vessels in the bleeding diverticulum. We experienced a case in which we succeeded in directly observing the exposed blood vessels of a sigmoid colon diverticulum using the ultrathin endoscope. The patient was a 71‐year‐old man who experienced rebleeding after hemostasis in the sigmoid colon by endoscopic band ligation. In the case of diverticular bleeding in the sigmoid colon, we showed that identifying exposed blood vessels by observing the diverticulum under direct vision using the ultrathin endoscope may be useful for hemostasis.

## INTRODUCTION

In recent years, cases of diverticular bleeding have become more common.^1^ Although identifying the bleeding diverticulum is difficult, it is even more difficult to identify the exposed blood vessels in the bleeding diverticulum. Colonic diverticula are too small for exposed blood vessels to be observed under direct vision using conventional endoscopy. We encountered a case in which hemostasis could be achieved after direct observation of the exposed blood vessel in the diverticulum of the sigmoid colon by using the ultrathin endoscopy.

## CASE REPORT

**FIGURE 1:**
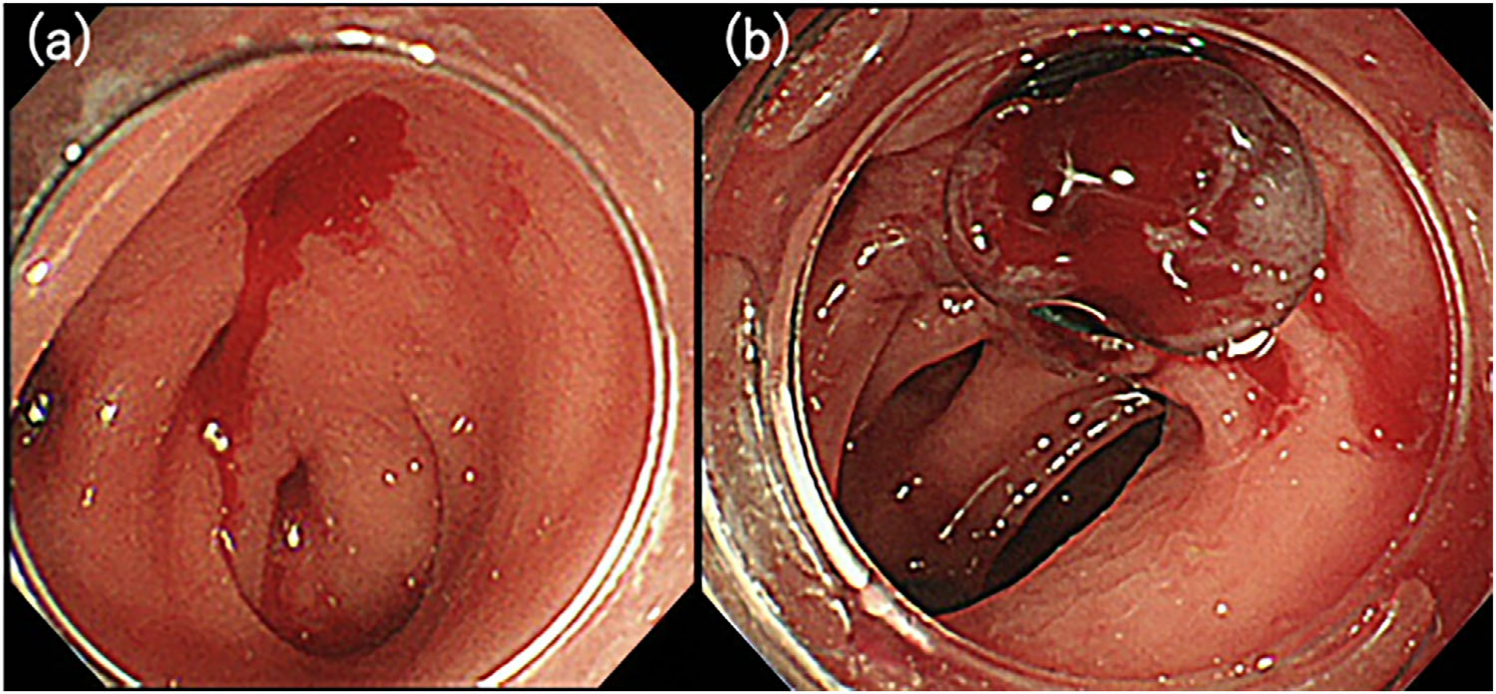
(a) Emergency colonoscopy was performed and diverticular bleeding was diagnosed in the sigmoid colon. (b) Hemostasis was achieved by endoscopic band ligation.

**FIGURE 2:**
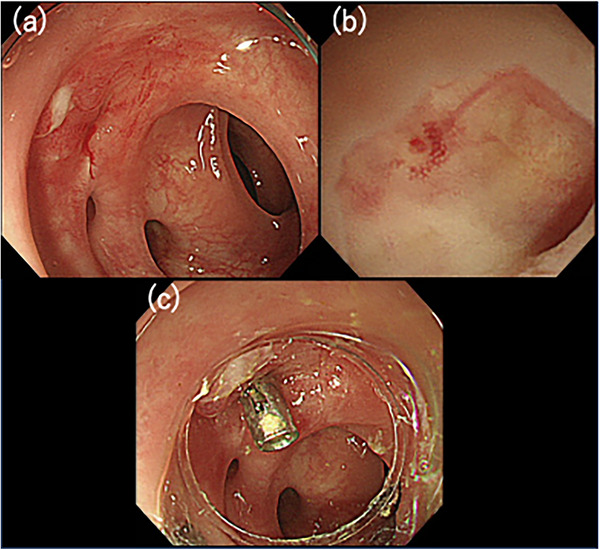
(a) Emergency colonoscopy was performed because of rebleeding. The O‐ring of the endoscopic band ligation had fallen off, and active bleeding was observed from the same diverticulum. Direct observation of the exposed blood vessel was not possible when using conventional endoscopy. (b) After the removal of the clot in the diverticulum, ultrathin endoscopy with a dedicated transparent hood was inserted into the target diverticulum. A pulsatile exposed blood vessel in the diverticulum could be identified. (c) Hemostasis was achieved using a clip. The patient was discharged without recurrence of bleeding.

The patient was a 71‐year‐old man who was urgently hospitalized for hematochezia. Diverticular bleeding in the sigmoid colon was diagnosed by emergency colonoscopy (Figure 1a). Hemostasis was achieved by endoscopic band ligation (EBL; Figure 1b). Two days after hemostasis, hematochezia was found again. A large amount of bloody stool was found from the rectum to the sigmoid colon. The O‐ring of the previous EBL had fallen off and active bleeding was observed from the same diverticulum (Figure 2a). An endoscopic observation was performed using the ultrathin endoscope (GIF‐XP260N; Olympus) with a dedicated transparent hood (Nichendo; Yasui Co., Ltd.), which enabled observation of the pulsatile exposed blood vessel in the diverticulum under direct vision (Figure 2b). The endoscope was then changed to the conventional endoscope (PCF‐H290I; Olympus, Tokyo, Japan) and hemostasis was achieved by direct clipping for the exposed blood vessel (Figure 2c).

## DISCUSSION

In recent years, hemostasis by EBL has been performed to treat diverticular bleeding.^2^ However, in the left side colon, including the sigmoid colon, the efficacy of EBL is low, and sometimes we experience rebleeding after EBL.^3^, ^4^ In that case, another option is clipping, and observation of the diverticulum using the ultrathin endoscope will contribute to clipping.^5^

## CONFLICT OF INTEREST STATEMENT

None.

## ETHICS STATEMENT

N/A

## PATIENT CONSENT STATEMENT

N/A

## CLINICAL TRIAL REGISTRATION

N/A

## Supporting information


Video S1 Direct observation of colonic diverticulum bleeding using an ultrathin endoscope.
