# Supplementary material for: Direct observation of an exposed blood vessel in a colonic diverticulum using ultrathin endoscopy
Source: DEN Open. 2024 Oct 29;5(1):e70032. doi: 10.1002/deo2.70032 (PMC11522026; doi:10.1002/deo2.70032)
Supplement: Supplementary file 1 — Video S1 Direct observation of colonic diverticulum bleeding using an ultrathin endoscope. [file DEO2-5-e70032-s001.docx]

Video S1 can be viewed here:

<https://drive.google.com/file/d/1CSCHXa-NyZEyvEdklaWfWfcz-fsWXcrH/view>
